# Impairment of exogenous lactate clearance in experimental hyperdynamic septic shock is not related to total liver hypoperfusion

**DOI:** 10.1186/s13054-015-0928-3

**Published:** 2015-04-22

**Authors:** Pablo Tapia, Dagoberto Soto, Alejandro Bruhn, Leyla Alegría, Nicolás Jarufe, Cecilia Luengo, Eduardo Kattan, Tomás Regueira, Arturo Meissner, Rodrigo Menchaca, María Ignacia Vives, Nicolas Echeverría, Gustavo Ospina-Tascón, Jan Bakker, Glenn Hernández

**Affiliations:** Departamento de Medicina Intensiva, Facultad de Medicina, Pontificia Universidad Católica de Chile, Marcoleta 367, Santiago, 8320000 Chile; Departamento de Cirugía Digestiva, Facultad de Medicina, Pontificia Universidad Católica de Chile, Marcoleta 367, Santiago, 8320000 Chile; Unidad de Pacientes Críticos, Hospital Clínico Universidad de Chile, Santos Dumont 999, Santiago, 8380000 Chile; Intensive Care Medicine Department, Fundación Valle del Lili - Universidad ICESI, Avenida Simón Bolívar Carrera 98, Cali, 76001000 Colombia; Department of Intensive Care Adults, Erasmus MC University Medical Centre, PO Box 2040, Room H625, Rotterdam, CA 3000 The Netherlands

## Abstract

**Introduction:**

Although the prognostic value of persistent hyperlactatemia in septic shock is unequivocal, its physiological determinants are controversial. Particularly, the role of impaired hepatic clearance has been underestimated and is only considered relevant in patients with liver ischemia or cirrhosis. Our objectives were to establish whether endotoxemia impairs whole body net lactate clearance, and to explore a potential role for total liver hypoperfusion during the early phase of septic shock.

**Methods:**

After anesthesia, 12 sheep were subjected to hemodynamic/perfusion monitoring including hepatic and portal catheterization, and a hepatic ultrasound flow probe. After stabilization (point A), sheep were alternatively assigned to lipopolysaccharide (LPS) (5 mcg/kg bolus followed by 4 mcg/kg/h) or sham for a three-hour study period. After 60 minutes of shock, animals were fluid resuscitated to normalize mean arterial pressure. Repeated series of measurements were performed immediately after fluid resuscitation (point B), and one (point C) and two hours later (point D). Monitoring included systemic and regional hemodynamics, blood gases and lactate measurements, and *ex-vivo* hepatic mitochondrial respiration at point D. Parallel exogenous lactate and sorbitol clearances were performed at points B and D. Both groups included an intravenous bolus followed by serial blood sampling to draw a curve using the least squares method.

**Results:**

Significant hyperlactatemia was already present in LPS as compared to sham animals at point B (4.7 (3.1 to 6.7) versus 1.8 (1.5 to 3.7) mmol/L), increasing to 10.2 (7.8 to 12.3) mmol/L at point D. A significant increase in portal and hepatic lactate levels in LPS animals was also observed. No within-group difference in hepatic DO_2_, VO_2_ or O_2_ extraction, total hepatic blood flow (point D: 915 (773 to 1,046) versus 655 (593 to 1,175) ml/min), mitochondrial respiration, liver enzymes or sorbitol clearance was found. However, there was a highly significant decrease in lactate clearance in LPS animals (point B: 46 (30 to 180) versus 1,212 (743 to 2,116) ml/min, *P* <0.01; point D: 113 (65 to 322) versus 944 (363 to 1,235) ml/min, *P* <0.01).

**Conclusions:**

Endotoxemia induces an early and severe impairment in lactate clearance that is not related to total liver hypoperfusion.

**Electronic supplementary material:**

The online version of this article (doi:10.1186/s13054-015-0928-3) contains supplementary material, which is available to authorized users.

## Introduction

The evolution of serum lactate levels during septic shock resuscitation represents a balance between aerobic or anaerobic production, and clearance by different tissues [1-3]. Clearance is more strictly a pharmacokinetic term used to describe drug or substance elimination from the body without identifying the elimination process. The term ‘lactate clearance’ has somehow been erroneously used in medical literature when referring to the process of change in serial lactate levels in response to therapy. Indeed, a decrease in serum lactate levels could be caused either by a decreased production or increased clearance, and the inverse is also true [1-3].

Although the liver plays a major role in systemic lactate clearance [1], persistent hyperlactatemia has only been related to impaired hepatic clearance in cases of severe shock with a comorbidity of liver ischemia or advanced cirrhosis [4,5]. Unfortunately, there is a large gap of knowledge in this area due to the relative lack of comprehensive physiological studies addressing the role of the liver in hyperlactatemia. In addition, current experimental and clinical studies provide conflicting data about liver lactate metabolism during sepsis, irrespective of the original source of lactate [6-16]. Douzinas *et al*. reported net lactate production by the liver in all of 10 stable septic patients with liver dysfunction [6], whereas De Backer *et al*. observed this in only 7% of 90 septic patients [7]. Levraut *et al*. evaluated whole body net lactate clearance and production in 34 stable septic patients with normal to mildly elevated lactate levels by a bolus infusion of L-lactate [9]. Patients with elevated baseline lactate levels exhibited approximately 50% lower lactate clearance. Others have stressed a dynamic relationship between arterial and/or portal lactate levels, and hepatic lactate uptake [17]. In addition, hepatic lactate metabolism might even be a saturable process [18], influenced by pH [19-21], substrate availability [13,22], and sepsis [1,11]. Part of these conflicting results could be explained by the extreme variability in the design of both experimental and clinical studies [6-16]. Nevertheless, common opinion suggests a significant role for hepatic dysfunction in delayed lactate clearance. If liver perfusion is shown to be a major component in delayed lactate clearance, this could have particularly significant clinical implications.

To address this, we designed a short-term controlled physiological study in a well-standardized sheep model of endotoxic shock, which characteristically evolves into a hyperdynamic hyperlactatemic profile after resuscitation [23]. Our objectives were to establish if endotoxemia impairs whole body net lactate clearance, and to explore a potential role for total liver hypoperfusion during the early phase of septic shock.

## Methods

The study was performed at the Medical Research Center of the School of Medicine, Pontificia Universidad Católica de Chile, as part of a major project exploring the influence of adrenergic stimulation and blockade over the determinants of lactate production and utilization in endotoxic shock (FONDECYT Chile grant number 1130200). The study was performed in accordance with the National Institutes of Health Guide for the Care and Use of Laboratory Animals, and with the approval of the *Comité de Etica y Bienestar Animal* of our University (CEBA 12-031).

### Anesthesia and surgery

#### Anesthesia

Sheep (weight: 29 to 41 kg) were received at the Research Center, and fasted for 24 hours before surgery, except for free water access. Sheep were premedicated with 20 mg/kg ketamine and 0.25 mg/kg midazolam intramuscularly. After inserting a peripheral intravenous line and injecting 30 mcg/kg fentanyl + 0.5 mg/kg atracurium + 1 mg/kg lidocaine intravenously, sheep were intubated and connected to mechanical ventilation. Anesthesia was maintained with a continuous infusion of 2 mg/ml midazolam, 20 mcg/ml fentanyl, and 20 mg/ml ketamine, set at 0.5 ml/kg/h during invasive procedures, and at 0.25 ml/kg/h thereafter until the end of the experiment.

Muscle paralysis was maintained with a continuous infusion of 0.25 mg/kg/h atracurium throughout the experiment. Animals were ventilated with a volume-controlled ventilator (Dräger Evita XL®, Lübeck, Germany) with 8 cm H_2_O end-expiratory pressure. The fraction of inspired oxygen (FiO_2_) was adjusted to keep oxygen tension (pO_2_) levels between 100 and 150 mmHg. Tidal volume was kept at 10 ml/kg and minute ventilation adjusted to maintain partial oxygen pressure (PaCO_2_ (partial pressure of carbon dioxide)) levels between 35 and 45 mmHg. Throughout the experiment, carbon dioxide was monitored with a mainstream end-tidal CO_2_ detector (Philips Medical Systems, Eindhoven, The Netherlands). Throughout the surgical procedure, the animals received normal saline at 10 ml/kg/h. The body temperature of the animals was kept at 38 ± 0.5°C.

#### Surgery

An 8Fr sheath was placed in the left and right external jugular veins for subsequent placement of a pulmonary artery catheter and a hepatic vein catheter, respectively (the latter was placed in an accurate position with intra-abdominal ultrasound guidance). The left femoral artery and vein were surgically exposed, and an arterial catheter and a three-lumen central venous catheter were inserted for blood pressure monitoring, sampling, and infusions. Then, a midline laparotomy was performed, followed by a gastrostomy to allow drainage of gastric contents and a splenic vein ligature. Afterwards, the hepatic artery and the portal vein were exposed, and an appropriate ultrasound flow probe (Transonic®, Ithaca NY, USA) was placed around both vessels to assess total hepatic flow. Another catheter was inserted into the portal vein and then the laparotomy was closed. After surgery, the saline infusion was reduced to 5 ml/kg/h and maintained till the end of the experiment.

### Measurements

Several measurements were performed at different time points as described below.

1. Hemodynamic data extraction: Femoral and pulmonary arterial, central venous, and pulmonary capillary wedge pressures were recorded with quartz pressure transducers displayed on a multi-modular monitor (Datex- Engström®, Madison WI, USA). All pressure transducers were calibrated and zeroed at mid-chest level and obtained at end-expiration. Cardiac output (CO, L/min) was measured by a thermodilution technique (mean value of three measurements, CO module, Datex-Engström®, Madison WI, USA). Central venous blood temperature was recorded from the thermistor in the pulmonary artery catheter. Heart rate was measured from the electrocardiogram. Hemodynamic data were recorded every 30 minutes.
2. Systemic and hepatosplanchnic oxygen delivery, consumption, and lactate handling: Arterial, portal, hepatic vein, mixed venous, and central venous gases were measured with a blood gas analyzer (i-Stat® bedside gas analyzer, Princeton NJ, USA). Total hepatic blood flow was recorded with an ultrasound flow probe involving the hepatic artery and portal vein (Transonic®, Ithaca NY, USA). Systemic and hepatic oxygen delivery, consumption, and extraction were calculated according to standard formulas, which are summarized in Additional file 1.
3. Lactate assessment: Lactate concentrations at each experimental time point obtained from arterial and venous blood samples were determined directly by lactate scout monitor (Senslab®, Leipzig, Germany). We performed the measurements in triplicate and averaged results.
4. Lactate clearance: This technique was performed twice during the experiment. A bolus infusion of sodium L-lactate (1 mmol/kg, in a 50 ml saline solution) was infused via a central venous catheter over 15 minutes. Arterial lactate blood samples were taken at baseline and 1, 3, 6, 9, 15, and 20 minutes after the lactate bolus, and measured with the lactate scout monitor in triplicate (Senslab, Leipzig, Germany). Clearance was later analyzed using the least squares method with semi-logarithmic coordinates [9].
5. Sorbitol clearance: This technique was performed twice during the study since it has been proposed to indirectly assess liver blood flow due to its very high first pass liver extraction [24]. A bolus of D-sorbitol (30 mg/kg, 5% solution in saline buffer) was infused via a central venous catheter over one minute. Arterial blood samples were taken at baseline and 1, 3, 9, and 15 minutes after infusion, and immediately frozen. Sorbitol levels were measured later by the enzymatic method coupled to formazan formation according to the manufacturer directions (Biovision®, Milpitas CA, USA). Assessment of sorbitol clearance was similar to that of lactate clearance [9]
6. Mitochondrial respiration: This technique was used to assess functional vitality of liver mitochondria at the end of the experiment. Liver samples were taken at the end of the experiment and immediately put on cold phosphate buffered saline. Complex I- and II-dependent respiration rates were measured using high-resolution respirometry (Oxygraph-2 k®; Oroboros Instruments, Innsbruck, Austria), and are expressed as pmol/second/mg whole protein content. Active respiration after addition of adenosine diphosphate (ADP) (State 3) was measured using glutamate/malate and succinate as substrates for complex I and II, respectively [25].

### Experimental procedure

**Figure 1:**
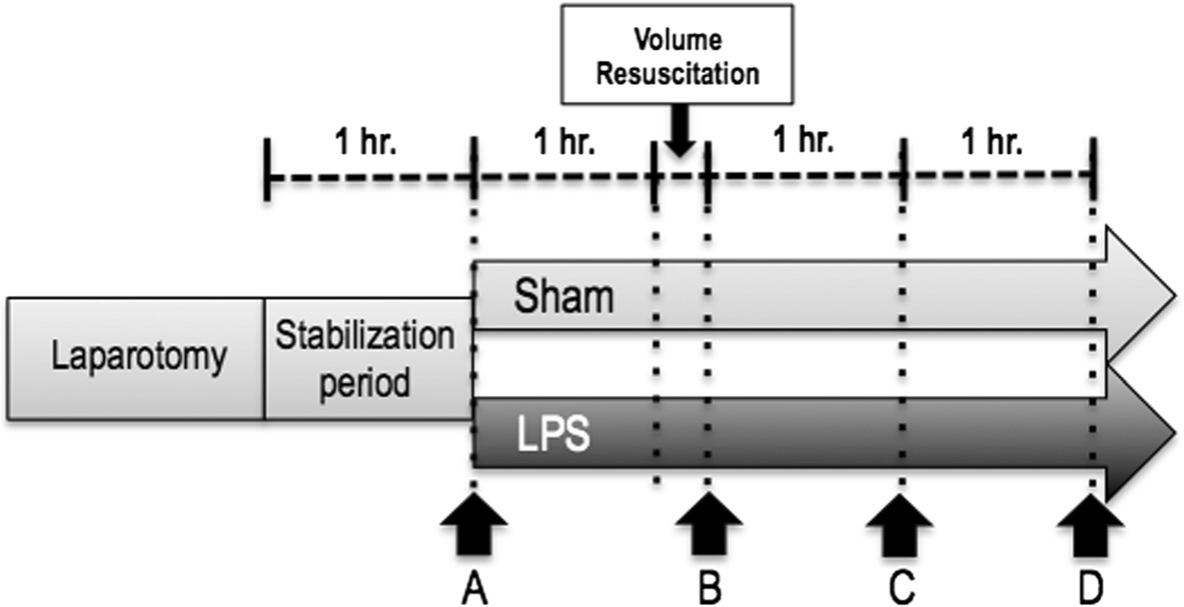
General scheme of the protocol. Complete hemodynamic, respiratory, and systemic and regional perfusion assessment was performed at points A, B, C, and D, except for lactate and sorbitol clearances that were done only at points A, B, and D. hr, hour; LPS, lipopolysaccharide.

After one hour of post-surgery stabilization, basal measurements were recorded (Figure 1, point A). Sheep were then alternatively assigned to either the endotoxin (lipopolysaccharide (LPS); n = 6) or sham (n = 6) group. In the protocol animals, sepsis was induced by a 5 mcg/kg endotoxin bolus (*Escherichia coli* 0111:β4; Sigma, St Louis MO, USA) given in 1 minute, followed by a continuous infusion of 4 mcg/kg/h during the rest of the experiment. Control animals were infused with saline. During the first hour of endotoxin or placebo infusion no other fluid was administered. Thereafter, resuscitation was performed with 5 ml/kg saline boluses repeated up to 3 times until a mean arterial pressure (MAP) target of 55 to 60 mmHg or a pulse pressure variation under 10% was achieved. If fluid loading failed to restore MAP to target levels norepinephrine was started. Repeated series of measurements were performed after fluid resuscitation (point B), and one and two hours later (Figure 1, points C and D, respectively).

At each time point hemodynamic and respiratory variables, blood temperature, hepatic blood flow, and arterial, mixed, central venous, portal, and hepatic vein blood gases and lactate samples were taken simultaneously. Samples for liver enzymes were taken, and sorbitol and lactate clearances performed at points A, B, and D. Following completion of the experiment, a liver biopsy for study of mitochondrial function was taken.

### Statistics

Non-parametric tests were used since data exhibited a non-normal distribution. Results are expressed as median and interquartile range. Intra-group medians were compared with Friedman’s test and Bonferroni’s *post-hoc* correction. Inter-group medians were compared by Mann–Whitney U test. SPSS software version 17.0 (Chicago, IL, USA) was used for statistical calculations. *P* <0.05 was considered as statistically significant. All reported *P* values are two-sided.

## Results

LPS induced a hemodynamic profile of septic shock with significant hypotension and pulmonary hypertension, and a trend of increased CO (Table 1). All LPS animals required norepinephrine in progressively higher doses up to 2.43 (2.2 to 2.7) mcg/kg/min at point D. A significant hyperlactatemia was already present in LPS as compared to sham animals (4.7 (3.1 to 6.7) versus 1.8 (1.5 to 3.7) mmol/L) following fluid resuscitation (point B), increasing to 10.2 (7.8 to 12.3) mmol/L at the end of the experiment (point D) (Table 2).

**Table 1:** **Hemodynamic and respiratory variables in sham versus LPS animals**

**Variable**	**Group**	**A**	**B**	**C**	**D**	***P*** ^**a**^
HR (bpm)	Sham	146 (111–169)	149 (117–180)	137 (122–161)	146 (127–159)	0.78
	LPS	133 (107–163)	152 (128–164)	147 (123–150)	127 (113–142)	0.12
MAP (mmHg)	Sham	81 (64–99)	85 (70–108)	87 (73–100)	68 (58–82)	0.13
	LPS	92 (79–103)	59 (56–80)^b^	57 (57–62)^b^	63 (56–75)	0.08
CO (L/min)	Sham	2.2 (1.9-2.6)	2.4 (2.2-2.9)	2.5 (2.1-3.0)	1.9 (1.6-2.6)	0.10
	LPS	2 (1.8-3.1)	3 (2.8-4.7)	3.1 (2.2-3.8)	2.8 (2.2-3.4)	0.18
MPAP (mmHg)	Sham	11 (10–13)	12 (10–13)	11 (10–112)	11 (8–11)	0.15
	LPS	13 (13–15)^b^	19 (14–22)^b^	18 (16–26)^b^	18 (16–29)^b^	0.07
PAOP (mmHg)	Sham	5 (4–6)	4.5 (4–5)	3.5 (3–6)	4 (3–5)	0.80
	LPS	8 (5–9)	8 (6–9)	8 (7–8)^b^	8 (7–8)^b^	0.56
Minute ventilation (L/min)	Sham	5.7 (5.2-6.4)	5.7 (4.8-6.4)	5.8 (4.8-6.6)	5.0 (4.4-6)	0.17
	LPS	5.9 (5.6-6.4)	6.2 (5.5-6.9)	6.3 (5.5-7.6)	6.5 (5.8-7.7)^b^	0.35
Plateau pressure (mmHg)	Sham	21 (18–22)	21 (20–23)	23 (19–25)	22 (21–23)	0.40
	LPS	19 (18–22)	24 (20–27)	24 (21–29)	25 (22–28)	0.02
RR (bpm)	Sham	16 (15–17)	16 (14–17)	16 (14–17)	16 (14–16)	0.80
	LPS	17 (16–19)	19 (16–19)	19 (16–22)	19 (16–22)	0.21
DO_2_ (ml O2/min)	Sham	260 (137–343)	290 (255–419)	270 (266–373)	214 (195–334)	0.06
	LPS	224 (203–337)	277 (235–364)	221 (167–358)	203 (158–290)	0.18
VO_2_ (ml O2/min)	Sham	97 (58–132)	99 (83–106)	94 (74–111)	85 (62–96)	0.06
	LPS	67 (54–86)	64 (56–75)	63 (53–76)	67 (59–85)	0.80
O_2_ER (%)	Sham	0.33 (0.25-0.37)	0.29 (0.25-0.4)	0.29 (0.25-0.41)	0.31 (0.26-0.47)	0.56
	LPS	0.28 (0.24-0.31)	0.24 (0.17-0.30)	0.30 (0.18-0.36)	0.35 (0.25-0.42)	0.17
SvO_2_ (%)	Sham	69 (65–74)	67 (63–74)	70 (60–75)	68 (55–75)	0.32
	LPS	77 (70–78)	76 (67–84)	68 (61–84)	65 (54–77)	0.09

CO, cardiac output; DO_2_, oxygen delivery; HR, heart rate; LPS, lipopolysaccharide; MAP, mean arterial pressure; MPAP, mean pulmonary arterial pressure; O_2_ER, oxygen extraction rate; PAOP, pulmonary artery occlusion pressure; RR, respiratory rate; SvO_2_, mixed venous oxygen saturation; VO_2_, oxygen consumption. Values are presented as median (interquartile range). *P* <0.05 considered as significant. ^a^Significant changes over time within groups. Comparison made with Friedman’s test and Bonferroni’s *post-hoc* correction. ^b^Significant difference between groups at the same time point. Comparison made with Mann–Whitney U test.

**Table 2:** **Evolution of serum lactate, sorbitol, and lactate clearances at different time points**

**Variable**	**Group**	**A**	**B**	**C**	**D**	***P*** ^**a**^
Arterial lactate (mmol/L)	Sham	2.4 (1.5-4.3)	1.8 (1.5-3.7)	2.2 (1.8-4.7)	2 (1.8-3.3)	0.8
	LPS	2.8 (2–3.6)	4.7 (3.1-6.7)^b^	7.1 (5.1-9.4)^b^	10.2 (7.8-12.3)^b^	0.01
Sorbitol clearance (ml/min)	Sham	582 (327–739)	385 (221–1,060)	NA	453 (348–612)	0.5
	LPS	481 (354–506)	519 (333–578)	NA	581 (318–824)	0.23
Lactate clearance (ml/min)	Sham	1,299 (418–2,187)	1212 (743–2,116)	NA	944 (363–1,235)	0.60
	LPS	1,066 (108–1,660)	46 (30–180)^b^	NA	113 (65–322)^b^	0.01

Values are presented as median (interquartile range). *P* <0.05 considered as significant. ^a^Significant changes over time within groups. Comparison made with Friedman’s test and Bonferroni’s *post-hoc* correction. ^b^Significant difference between groups at the same time point. Comparison made with Mann–Whitney U test. LPS, lipopolysaccharide.

Portal and hepatic vein lactate levels were significantly higher in the LPS animals at different time points and tended to increase over time (Table 3). Analysis of portohepatic vein lactate differences showed very low extraction rates (point B: portal lactate 4.0 and hepatic vein lactate 4.1 mmol/L; point C: portal lactate 6.4, hepatic vein lactate 6.1 mmol/L; point D: portal lactate 8.3, hepatic vein lactate 7.3 mmol/L; Table 3).

**Table 3:** **Evolution of hepatosplanchnic flow and perfusion parameters at different time points**

**Variable**	**Group**	**A**	**B**	**C**	**D**	***P*** ^**a**^
SpO_2_ (%)	Sham	86 (74–90)	86 (76–91)	79 (76–85)	78 (74–89)	0.64
	LPS	85 (75–91)	81 (75–86)	76 (65–85)	66 (53–72)^b^	0.08
ShO_2_ (%)	Sham	73 (62–87)	69 (65–80)	75 (64–81)	72 (55–84)	0.43
	LPS	77 (69–80)	75 (72–84)	71 (63–73)	68 (46–80)	0.25
Portal lactate (mmol/L)	Sham	1.2 (0.6-2.2)	2.2 (1.1-3.8)	1.8 (0.9-3.5)	1.3 (1.1-2.9)	0.31
	LPS	2.6 (2.0-3.8)^b^	4 (3.3-6.3)^b^	6.4 (5.8-8.2)^b^	8.3 (7–9.5)^b^	0.06
Hepatic lactate (mmol/L)	Sham	1.3 (0.6-3.1)	1.9 (0.8-3.8)	1.6 (1.1-2.8)	1.2 (1–2.5)	0.52
	LPS	2.1 (1.6-2.9)	4.1 (3.3-6.3)^b^	6.1 (5.1-7.7)^b^	7.3 (6.4-8.6)^b^	0.04
Hepatic DO_2_ (ml O_2_/min)	Sham	117 (53–150)	107 (50–160)	93 (55–128)	88 (42–150)	0.10
	LPS	98 (68–133)	97 (75–110)	76 (52–88)	56 (36–70)	0.03
Hepatic VO_2_ (ml O_2_/min)	Sham	34 (16–49)	28 (13–45)	34 (10–41)	38 (13–41)	0.21
	LPS	20 (11–34)	24 (20–30)	28 (17–37)	22 (19–25)	0.7
Hepatic O_2_ER (%)	Sham	0.33 (0.25-0.37)	0.33 (0.19-0.45)	0.46 (0.30-058)	0.34 (0.22-0.59)	0.81
	LPS	0.25 (0.10-0.35)	0.22 (0.20-0.34)	0.37 (0.32-0.44)	0.42 (0.30-0.73)	0.34
Total hepatic blood flow (ml/min)	Sham	707 (585–1,335)	770 (660–1,250)	732 (673–1,213)	655 (593–1,175)	0.40
	LPS	1,150 (753–1,568)	1,275 (1,181-1,700)	1,050 (880–1,227)	915 (773–1,046)	0.048
Fractional hepatic blood flow (%)	Sham	47 (32–62)	41 (34–47)	39 (30–48)	46 (35–57)	0.43
	LPS	50 (43–56)	41 (39–44)	35 (31–43)	32 (26–43)^b^	0.08

Hepatic DO_2_, hepatic oxygen delivery; Hepatic O_2_ER, hepatic oxygen extraction rate; Hepatic VO_2_, hepatic oxygen consumption; ShO_2_, hepatic vein oxygen saturation; SpO_2_, portal vein oxygen saturation.

Values are presented as median (interquartile range). *P* <0.05 considered as significant. ^a^Significant changes over time within groups. Comparison made with Friedman’s test and Bonferroni’s *post-hoc* correction. ^b^Significant difference between groups at the same time point. Comparison made with Mann–Whitney U test.

When comparing intergroup values at any time point, no differences in hepatic DO_2_, VO_2_, and O_2_ extraction, total hepatic blood flow, or in hepatic vein or portal oxygen saturations were detected, except for portal O_2_ saturation at point D (Table 3). However, hepatic DO_2,_ total hepatic blood flow, and fractional hepatic blood flow tended to decrease over time in LPS animals.

Aminotransferases, bilirubin (Br), and gamma-glutamyl transferase (GGT) were comparable between sham and LPS animals at the end of the experiment (alanine aminotransferase (ALT): 16 (15 to 17) versus 20 (15 to 30) U/L; aspartate aminotransferase (AST): 144 (124 to 162) versus 264 (204 to 277) U/L; Br: 0.09 (0.07 to 0.09) versus 0.09 (0.08 to 0.1) mg/dl; GGT: 49 (39 to 51) versus 64 (50 to 83) U/L). Mitochondrial respiration was not different between groups (basal: sham 66 (47 to 88), LPS 27 (17 to 41); complex I: sham 450 (113 to 524), LPS 206 (160 to 248); complex I + II: sham 644 (324 to 719), LPS 350 (304 to 471) pmol/sec/mg).

**Figure 2:**
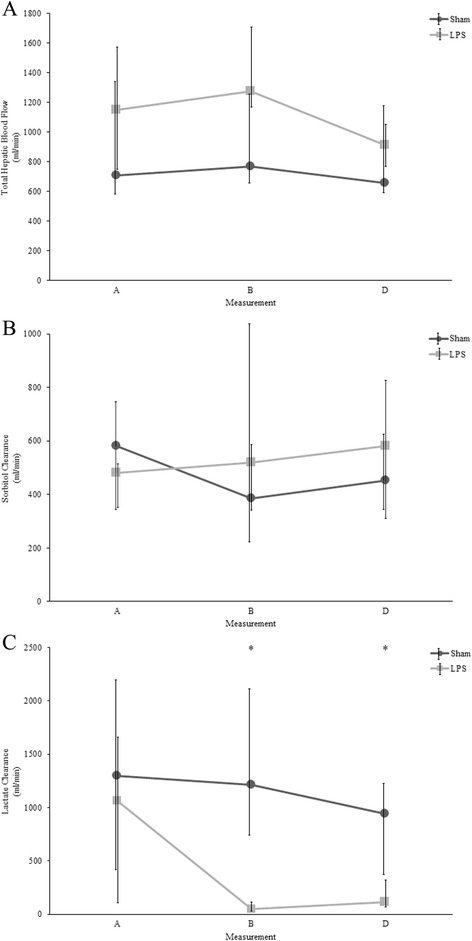
Evolution of total hepatic blood flow. **(A)**, sorbitol **(B)** and lactate clearances **(C)** at different time points in sham versus LPS animals. As shown, no differences were detected in flow or sorbitol clearances, but there was a highly significant decrease in lactate clearance in LPS animals at points B and D. Bars represent median (interquartile range). *P <0.05, comparison made with Mann–Whitney U test. LPS, lipopolysaccharide.

Sorbitol clearance at points A, B, and D was preserved in both LPS and sham animals. Following the induction of shock (point B), lactate clearance decreased significantly in the LPS animals without recovery at the end of the experiment (point D, Table 2, Figure 2).

## Discussion

Our results demonstrate that endotoxic shock induces a very early and severe impairment in exogenous whole body net lactate clearance that is not related to total liver hypoperfusion or evident biochemical dysfunction. However, the very low portohepatic vein lactate differences suggest at least a liver metabolic inability to handle increased lactate loads. This appears to be a specific rather than generalized metabolic dysfunction in LPS animals as the clearance of sorbitol, a polyol molecule with a 96% first pass liver extraction, was preserved. This finding might lead to reconsider the role of the liver in persistent hyperlactatemia, and opens new perspectives for translational and clinical research on a complex potential resuscitation target, such as lactate.

To assess lactate clearance we used the method proposed by Levraut *et al*. [9]. Whole body net lactate clearance and lactate production were evaluated in 34 stable septic patients with normolactatemia or mild hyperlactatemia by modeling the lactate kinetics induced by an exogenous lactate bolus. They found that hyperlactatemic as compared to normolactatemic patients exhibited approximately 50% lower lactate clearance after the challenge. In the current study, lactate clearance decreased by more than 90% in LPS animals. This dramatic decrease in clearance was observed very early after LPS administration (a mean of 80 minutes after initial bolus of LPS) and was not restored by systemic resuscitation. Thus, impaired lactate clearance develops very early in septic shock, varies in severity according to the magnitude of the septic insult, and persists over time considering that 12 out of 34 patients in Levraut’s study [9] were studied after three days of septic shock. Additionally, liver biochemistry was almost normal in both studies, suggesting a selective metabolic dysfunction not easily recognizable by systemic hemodynamics or common liver functional tests.

The liver is the primary organ involved in lactate clearance, accounting for approximately 50% of uptake [1]. L-lactate is taken up by the hepatocyte mainly via a monocarboxylate carrier, and it is metabolized within the hepatocytes by oxidation or as a substrate for neoglucogenesis. Total hepatic blood flow is critical in this process, but the relationship between flow and lactate clearance is controversial [6,7,9,16]. Although hepatic lactate extraction decreases during low flow states without sepsis, this occurs only after dramatic decreases in flow [17,26,27]. Samsel *et al*. studied eight pump-perfused canine livers and demonstrated that lactate clearance decreases only when a critical O_2_ delivery is reached [17]. Iles *et al*. observed that lactate uptake decreases rapidly when blood flow falls below 25% of normal in an isolated rat liver model [26]. Tashkin *et al*. confirmed that this does not occur under moderate flow reductions where hepatic lactate uptake can respond with a compensatory increase [27].

Although under normal conditions there is a large metabolic reserve in hepatic lactate metabolism that may compensate for moderate reductions in flow as stated above, this may not be the case in pathological conditions such as sepsis. Chrusch *et al*. observed that lactate extraction after an exogenous lactate loading was only 3.6% in septic animals as compared to 14.9% in non-septic controls [19]. Pscheidl *et al*. used radioactive microspheres to assess changes in regional blood flow during low-dose LPS infusion in a rat model [28]. They found decreased portal flow that was compensated by the hepatic arterial buffer response, thus maintaining total hepatic flow. Nevertheless, lactate clearance decreased significantly despite this preserved flow [28]. Santak *et al*. found a persistent increase in total hepatic flow in LPS versus control animals in a long-term pig endotoxin shock model, but lactate clearance decreased progressively for up to 24 hours [16]. Our results are in line with these previous studies, with some new aspects highlighted below.

However, there are some contrasting data. Severin *et al*. applied a bolus LPS dose of 6 mcg/kg in a chronically catheterized rat model [15]. The hemodynamic effect was modest, with a minor increase in lactate from 0.84 to 2.7 mmol/L at 120 minutes. Interestingly, the liver increased lactate extraction as suggested by a negative shift in the hepatic vein to portal vein lactate concentration gradient [15]. Bellomo *et al*. found no correlation between hepatic lactate uptake and liver oxygen transport, consumption, or extraction in a dog endotoxic model [11]. Creteur *et al*. performed one of the most relevant studies in 18 anesthetized dogs receiving a very high dose of LPS [29]. Twelve of the animals were subjected to a dynamic cardiac tamponade to induce a hypodynamic state with progressive reductions in hepatic blood flow. These animals experienced decreased liver lactate extraction below a critical oxygen delivery rate. The other six septic dogs experienced increased gut lactate production, but this was compensated by a huge increase in liver lactate uptake, and thus whole hepatosplanchnic lactate production remained stable [29].

Eventually, these confounding data may be explained by the extreme heterogeneity of experimental studies addressing lactate handling during septic shock in terms of model (intravenous bolus, intraperitoneal, or continuous infusion of LPS; or cecal ligation and puncture), observation periods, resuscitation strategies, and techniques to assess flow, among others. Very few models have reproduced the characteristic post-resuscitative hyperdynamic septic shock in humans as in our study.

Hepatosplanchnic hypoperfusion has been traditionally considered as an important mechanism during septic shock leading to bacterial or endotoxin translocation, and to impairment of several aspects of liver function, among other consequences [12]. However, regional perfusion is difficult to assess, even in the experimental setting. In our study we complemented direct flow measurements, O_2_ delivery, and extraction assessments, with D-sorbitol clearance [24]. D-sorbitol has a high liver extraction ratio and its clearance is dependent on delivery to the hepatocytes by hepatic blood flow. Therefore, the rate of plasma clearance of sorbitol can be used as a measure of liver blood flow and function [24]. In our experiment, we did not find hepatic hypoperfusion or hypoxia, as total hepatic blood flow, sorbitol clearance, hepatic DO_2_, VO_2_ and O_2_ extraction, hepatic vein oxygen saturation, and liver O_2_ extraction, *ex-vivo* mitochondrial respiration, and liver enzymes, such as aminotransferases normally released during hepatic ischemia, were comparable between LPS and sham groups. Only at the end of the experiment did hepatic blood flow and eventually mitochondrial respiration decrease in LPS animals. Therefore, we cannot rule out that hypoperfusion may play a role in a more prolonged model. However, the highly significant impairment in clearance as early as at point B without a significant change in total hepatic blood flow at this point precludes hypoperfusion from being an important factor, at least in early stages after fluid resuscitation.

Our study introduced new methodological aspects such as a more exhaustive assessment of potential overt or occult liver hypoperfusion, a more clearly defined resuscitation protocol, and simultaneous evaluation of lactate and sorbitol clearances. In this study, the decrease in whole body net lactate clearance was clearly out of proportion with systemic and regional hemodynamics, and was present in absence of traditional biochemical evidence of liver injury or other metabolic dysfunctions, such as with sorbitol uptake. However, the very low portohepatic vein lactate differences suggest at least a liver metabolic inability to handle increased lactate loads, and thus an acquired metabolic dysfunction or shift. Thus, it appears that a relevant specific metabolic liver dysfunction without clear clinical expression can occur during septic shock resuscitation even with preserved hemodynamics, and this could have a significant impact on lactate clearance.

Several factors could explain these findings. First, several studies have suggested that endotoxin or pro-inflammatory mediators may inactivate pyruvate dehydrogenase or phosphoenolpyruvate carboxykinase enzymes, thus potentially blocking two metabolic pathways for lactate metabolism [30,31]. Second, hepatic lactate uptake might be a saturable process with second order kinetics [18]. Therefore, repeated exogenous lactate loading over a short period could lead to lactate accumulation by saturating metabolic clearance. However, the same loading in sham animals did not produce any accumulation, suggesting that a metabolic dysfunction should be present for this to occur. Third, impairment in hepatic microcirculation might be implicated [32-35]. Doubtlessly, severe liver microcirculatory abnormalities can be found in the presence of normal total hepatic flow. Either portal or hepatic arterial LPS injection leads to a rapid and transient increase in intrahepatic resistances at the presinusoidal level [31-35]. Redistribution of intrahepatic blood flow in concert with a complex interplay between sinusoidal endothelial cells, liver macrophages, and passing leukocytes, leads to a decreased perfusion and blood flow velocity in the liver sinusoids [31-35]. Activation and dysfunction of the endothelial cell barrier, together with abnormalities in the nitric oxide pathway, with subsequent invasion of neutrophils and formation of microthrombi may further enhance liver tissue ischemia and damage [31-35]. Unfortunately, we did not specifically assess hepatic microcirculation. However, preserved liver VO_2_ and normality of *ex-vivo* mitochondrial respiration, taken together with the absence of positive portohepatic vein lactate gradients, makes it unlikely that this phenomena had some impact in lactate clearance. Fourth, local production of lactate by activated immune cells in the liver during sepsis is another biologically plausible cofactor [11,36]. Theoretically, the impact on local lactate production could be proportional to the inflammatory stimulus, and thus might change according to the experimental model selected. Unfortunately, our study design only allowed us to address some of these potential mechanisms, but we were able to rule out liver hypoperfusion or a generalized metabolic dysfunction.

We acknowledge several limitations of our study. First, we did not assess renal uptake of lactate, which is an important omission since the kidneys are second only to the liver in lactate clearance after an external lactate load [1]. Second, we did not quantitatively assess metabolic handling by the liver. Third, we did not assess hepatic microcirculation. Fourth, repeated lactate loads over a short period of time may have saturated liver metabolic capacity, thus introducing bias. However, the strengths of the study shows that we reproduced a hyperdynamic septic shock in a large animal model closely resembling human cases. We assessed liver perfusion more comprehensively than previous studies and can effectively rule out the presence of hypoperfusion or hypoxia. We also demonstrated a severe impairment of lactate clearance very early after endotoxin infusion, despite normal clinical liver biochemistry and other metabolic functions. Finally, we demonstrated that this process persists over time, even while maintaining systemic hemodynamics. Of course, our findings cannot be extrapolated to low flow states or hypodynamic shock, where liver hypoperfusion can induce a decrease in lactate clearance.

The results of our study might have relevant clinical implications. Although tissue hypoperfusion has been traditionally considered an important cause of persistent hyperlactatemia, there is increasing evidence for concomitant non-hypoxic, and thus, non-flow-dependent mechanisms such as hyperadrenergia or subclinical sepsis-induced liver metabolic dysfunction, that may influence the time course of lactate recovery rate [2]. The distinction between these two scenarios should strongly impact the therapeutic approach. As an example, treatment of the latter with sustained efforts aimed at increasing DO_2_ could lead to severe resuscitation toxicity expressed as fluid overload, pulmonary edema, or intraabdominal hypertension, with no therapeutic benefit if the impaired lactate clearance is caused by a non-hypoxic liver dysfunction irrespective of the involved intimate pathogenic mechanisms. This is true even if lactate clearance is a saturable biochemical process. These considerations should not lead to a therapeutic nihilism concerning lactate. Hyperlactatemia is still a very strong prognostic factor. The above statements only pretend to highlight the relevance of trying to establish the cause of the problem before automatically prescribing fluids or additional resuscitation, since not all causes of hyperlactatemia are flow-sensitive [2,37].

## Conclusions

In conclusion, our results demonstrate that hyperdynamic endotoxic shock induces an early, and severe, impairment in lactate clearance that is not related to total liver hypoperfusion or evident biochemical dysfunction. This finding might have relevant clinical implications and should lead to further research to clarify the role of the liver in lactate handling during septic shock resuscitation.

## Key messages

- Endotoxemia induces an early and severe impairment in exogenous whole body net lactate clearance.
- This profound alteration is not related to liver hypoperfusion or other evident biochemical dysfunction.

## Additional file

Additional file 1:
**Most relevant hemodynamic formulas.**

## Abbreviations

ADP: Adenosine diphosphate
ALT: Alanine aminotransferase
AST: Aspartate aminotransferase
Br: Bilirubin
CO: Cardiac output
CO_2_: Carbon dioxide
DO_2_: Systemic oxygen delivery
FiO_2_: Fraction of inspired oxygen
GGT: Gamma-glutamyl transpeptidase
LPS: Lipopolysaccharide
MAP: Mean arterial pressure
pO_2_: Oxygen tension
VO_2_: Systemic oxygen consumption

## References

[1] Garcia-Alvarez M, Marik P, Bellomo R (2014). Sepsis-associated hyperlactatemia. Crit Care.

[2] Hernandez G, Bruhn A, Castro R, Regueira T (2012). The holistic view on perfusion monitoring in septic shock. Curr Opin Crit Care.

[3] Levy B (2006). Lactate and shock state: the metabolic view. Curr Opin Crit Care.

[4] Mizock BA (2001). The hepatosplanchnic area and hyperlactatemia: a tale of two lactates. Crit Care Med.

[5] Jeppesen JB, Mortensen C, Bendtsen F, Moller S (2013). Lactate metabolism in chronic liver disease. Scand J Clin Lab Invest.

[6] Douzinas EE, Tsidemiadou PD, Pitaridis MT, Andrianakis I, Bobota-Chloraki A, Katsouyanni K (1997). The regional production of cytokines and lactate in sepsis-related multiple organ failure. Am J Respir Crit Care Med.

[7] De Backer D, Creteur J, Silva E, Vincent JL (2001). The hepatosplanchnic area is not a common source of lactate in patients with severe sepsis. Crit Care Med.

[8] Barthelmes D, Jakob SM, Laitinen S, Rahikainen S, Ahonen H, Takala J (2010). Effect of site of lactate infusion on regional lactate exchange in pigs. Br J Anaesth.

[9] Levraut J, Ciebiera JP, Chave S, Rabary O, Jambou P, Carles M (1998). Mild hyperlactatemia in stable septic patients is due to impaired lactate clearance rather than overproduction. Am J Respir Crit Care Med.

[10] Hernandez G, Regueira T, Bruhn A, Castro R, Rovegno M, Fuentealba A (2012). Relationship of systemic, hepatosplanchnic, and microcirculatory perfusion parameters with 6-hour lactate clearance in hyperdynamic septic shock patients: an acute, clinical-physiological, pilot study. Ann Intensive Care.

[11] Bellomo R, Kellum JA, Pinsky MR (1996). Transvisceral lactate fluxes during early endotoxemia. Chest.

[12] Pastor CM, Billiar TR, Losser MR, Payen DM (1995). Liver injury during sepsis. J Crit Care.

[13] Revelly JP, Tappy L, Martinez A, Bollmann M, Cayeux MC, Berger MM (2005). Lactate and glucose metabolism in severe sepsis and cardiogenic shock. Crit Care Med.

[14] Michaeli B, Martinez A, Revelly JP, Cayeux MC, Chioléro RL, Tappy L (2012). Effects of endotoxin on lactate metabolism in humans. Crit Care.

[15] Severin PN, Uhing MR, Beno DW, Kimura RE (2002). Endotoxin-induced hyperlactatemia results from decreased lactate clearance in hemodynamically stable rats. Crit Care Med.

[16] Santak B, Radermacher P, Adler J, Iber T, Rieger KM, Wachter U (1998). Effect of increased cardiac output on liver blood flow, oxygen exchange and metabolic rate during long-term endotoxin-induced shock in pigs. Br J Pharmacol.

[17] Samsel RW, Cherqui D, Pietrabissa A, Sanders WM, Roncella M, Emond JC (1991). Hepatic oxygen and lactate extraction during stagnant hypoxia. J Appl Physiol.

[18] Naylor JM, Kronfeld DS, Freeman DE, Richardson D (1984). Hepatic and extrahepatic lactate metabolism in sheep: effects of lactate loading and pH. Am J Physiol.

[19] Chrusch C, Bautista E, Jacobs HK, Light RB, Bose D, Duke K (2002). Blood pH level modulates organ metabolism of lactate in septic shock in dogs. J Crit Care.

[20] Sestoft L, Marshall MO (1988). Regulation of lactate uptake and lactate production in liver from 48-h-starved rats: effects of pH, flow and glucose concentration. Clin Sci (Lond).

[21] Goldstein PJ, Simmons DH, Tashkin DP (1972). Effect of acid–base alterations on hepatic lactate utilization. J Physiol.

[22] Eldridge FL, T’So L, Chang H (1974). Relationship between turnover rate and blood concentration of lactate in normal dogs. J Appl Physiol.

[23] Dubin A, Edul VS, Pozo MO, Murias G, Canullán CM, Martins EF (2008). Persistent villi hypoperfusion explains intramucosal acidosis in sheep endotoxemia. Crit Care Med.

[24] Gommers D (2010). Noninvasive functional liver blood flow measurement: comparison between bolus dose and steady-state clearance of sorbitol in a small-rodent model. Am J Physiol Gastrointest Liver Physiol.

[25] Regueira T, Bänziger B, Djafarzadeh S, Brandt S, Gorrasi J, Takala J (2008). Norepinephrine to increase blood pressure in endotoxaemic pigs is associated with improved hepatic mitochondrial respiration. Crit Care.

[26] Iles RA, Baron PG, Cohen RD (1979). The effect of reduction of perfusion rate on lactate and oxygen uptake, glucose output and energy supply in the isolated perfused liver of starved rats. Biochem J.

[27] Tashkin DP, Goldstein PJ, Simmons DH (1972). Hepatic lactate uptake during decreased liver perfusion and hyposemia. Am J Physiol.

[28] Pscheidl EM, Wan JM, Blackburn GL, Bistrian BR, Istfan NW (1992). Influence of omega-3 fatty acids on splanchnic blood flow and lactate metabolism in an endotoxemic rat model. Metabolism.

[29] Creteur J, De Backer D, Sun Q, Vincent JL (2004). The hepatosplanchnic contribution to hyperlactatemia in endotoxic shock: effects of tissue ischemia. Shock.

[30] Hill M, McCallum R (1991). Altered transcriptional regulation of phosphoenolpyruvate carboxykinase in rats following endotoxin treatment. J Clin Invest.

[31] Vary TC, Siegel JH, Tall BD, Morris JG (1988). Metabolic effects of partial reversal of pyruvate dehydrogenase activity by dichloroacetate in sepsis. Circ Shock.

[32] La Mura V, Pasarín M, Rodriguez-Vilarrupla A, García-Pagán JC, Bosch J, Abraldes JG (2014). Liver sinusoidal endothelial dysfunction after LPS administration: a role for inducible-nitric oxide synthase. J Hepatol.

[33] Morel J, Li JY, Eyenga P, Meiller A, Gustin MP, Bricca G (2013). Early adverse changes in liver microvascular circulation during experimental septic shock are not linked to an absolute nitric oxide deficit. Microvasc Res.

[34] Ring A, Stremmel W (2000). The hepatic microvascular responses to sepsis. Semin Thromb Hemost.

[35] Spapen H (2008). Liver perfusion in sepsis, septic shock, and multiorgan failure. Anat Rec (Hoboken).

[36] McDonald B, Urrutia R, Yipp BG, Jenne CN, Kubes P (2012). Intravascular neutrophil extracellular traps capture bacteria from the bloodstream during sepsis. Cell Host Microbe.

[37] Hernandez G, Luengo C, Bruhn A, Kattan E, Friedman G, Ospina-Tascon GA (2014). When to stop septic shock resuscitation: clues from a dynamic perfusion monitoring. Ann Intensive Care.

