# Supplementary material for: Impairment of exogenous lactate clearance in experimental hyperdynamic septic shock is not related to total liver hypoperfusion
Source: Crit Care. 2015 Apr 22;19(1):188. doi: 10.1186/s13054-015-0928-3 (PMC4432956; doi:10.1186/s13054-015-0928-3)
Supplement: Additional file 1: — Most relevant hemodynamic formulas. [file 13054_2015_928_MOESM1_ESM.docx]

**Additional file 1**

**Hemodynamic formulas**

| Systemic oxygen delivery (DO_2_) = CO(L/min) * CaO_2_(ml/1000ml blood) *10 |
| --- |
| Systemic oxygen consumption (VO_2_) = CO(L/min) * CvO_2_(ml/1000ml blood) *10 |
| Oxygen-Extraction Ratio (O2ER) (%) = VO_2_/ DO_2_ |
| CaO_2_ (ml/1000 ml blood) = (1.34*Hb (gr/100ml)*O_2_ saturation of Hb) + (0.0031 *PaO_2_) |
| CvO_2_ (ml/1000 ml blood)= (1.34*Hb*(gr/100ml) O_2_ saturation of Hb) + (0.0031 *PvO_2_) |
| Hepatic DO_2_ (ml/min) = (Hepatic artery flow * CaO_2_)+(portal vein flow * portal vein O_2_ content) |
| Hepatic VO_2_ (ml/min) = (Hepatic DO_2_) - (Total hepatic blood flow * hepatic vein O_2_ content) |
| Hepatic O_2_ ER(%) hepatic VO_2_/DO_2_ |
| Fractional hepatic blood flow (%) = Total hepatic blood flow (ml/min) /CO (ml/min) |
|  |
|  |
